# Rapid and selective gut microbiome modulation by polyherbal formulation in type 2 diabetes

**DOI:** 10.1530/EC-25-0463

**Published:** 2026-01-09

**Authors:** Sintija Sauša, Annija Zodāne, Somit Kumar, Jānis Plūme, Jana Baranova, Tatjana Kozlova, Uģis Klētnieks, Harijs Saušs, Jānis Kloviņš, Valdis Pīrāgs, Kakarla Sai Mitravinda, Svjatoslavs Kistkins, Monta Brīvība

**Affiliations:** ^1^ Pauls Stradiņš Clinical University Hospital, Riga, Latvia; ^2^ University of Latvia, Riga, Latvia; ^3^ Latvian Biomedical Research and Study Centre, Riga, Latvia; ^4^ AVP Research Foundation, Coimbatore, India; ^5^ Longevity Alliance Baltic, Riga, Latvia; ^6^ Riga Technical University, Riga, Latvia

**Keywords:** gut microbiome, metformin, polyherbal formulation, type 2 diabetes, randomized-controlled trial

## Abstract

**Background:**

Metformin, the first-line treatment for type 2 diabetes, often induces gastrointestinal side effects, affecting treatment adherence. Recent research suggests that the gut microbiome mediates both the efficacy and tolerability of metformin. This study evaluates the effect of a polyherbal formulation, used as an add-on to metformin, on the gut microbiota in patients with type 2 diabetes and metformin intolerance.

**Methods:**

We report preliminary findings from the first 7-day intervention phase of an ongoing randomized, placebo-controlled, crossover trial (NCT06846138) in 27 adults with type 2 diabetes. Participants received either polyherbal formulations or a placebo alongside metformin for 7 days. Stool samples were collected pre- and post-intervention for shotgun metagenomic sequencing. Microbial diversity, composition, and pathway functions were analyzed using Kraken2, Bracken, and HUMAnN3. Continuous glucose monitoring was used to assess glycemic metrics.

**Results:**

No significant alpha-diversity changes were observed; however, beta-diversity differed significantly between arms (PERMANOVA *R*^2^ = 0.04, *P* = 0.04). In the polyherbal formulation group, 17 species changed post-treatment (FDR < 0.25), with significant increases in six *Bifidobacterium* spp. (e.g., *B. adolescentis*, *B. ruminantium*). In contrast, the placebo group showed no major microbial shifts. Polyherbal formulation also altered ten microbial pathways (FDR < 0.25). Continuous glucose monitoring revealed no short-term changes in glycemic levels.

**Conclusion:**

Short-term polyherbal formulation co-administration significantly modulates gut microbiota, promoting beneficial taxa, such as *Bifidobacterium* in metformin-treated type 2 diabetes patients. This supports the potential role of the polyherbal formulation in microbiome-targeted strategies to improve metformin tolerability and effectiveness.

## Introduction

Metformin is globally recommended as the first-line therapy for type 2 diabetes (T2D) due to its efficacy, cardioprotective benefits, and affordability. However, up to 30% of patients experience gastrointestinal (GI) side effects, such as bloating and diarrhea, leading to poor adherence and premature treatment discontinuation (1, 2). Growing evidence implicates the gut microbiome in metformin’s pharmacodynamics, as microbial shifts have been associated with both therapeutic response and side effect profiles (3, 4). We have previously reported clear and immediate gut microbiome alterations following metformin treatment in both healthy individuals and T2D patients (5, 6). In the well-characterized OPTIMED cohort of treatment-naïve T2D patients, we observed distinct microbial shifts already within 7 days of therapy. A decrease of *Clostridium bartlettii* and *Barnesiella intestinihominis* was observed, while *Parabacteroides distasonis* and *Oscillibacter* were increased (6). Furthermore, microbiome profiles differed by severity of metformin-induced GI side effects, with *Streptococcus parasanguinis* enriched in severe cases and *Ruminococcus lactaris* in those with none or mild symptoms (5). Metformin use in the literature has also been linked to increased levels of *Escherichia coli* and *Akkermansia muciniphila*, and decreased levels of *Intestinibacter*, changes that may underlie both therapeutic effects and adverse events (7, 8).

Co-administration of probiotics, such as *Bifidobacterium bifidum* G9-1, with metformin has been shown to reduce GI side effects. Moreover, fecal microbiota transplantation from metformin-treated donors has been shown to improve glucose tolerance in germ-free mice (5, 9), and metagenomic studies have suggested that the baseline composition of the gut microbiota may predict both efficacy and tolerability of metformin (10).

Alongside probiotics, prebiotics are also widely explored for managing GI dysbiosis (11, 12). In this context, polyherbal formulations (PHFs) have emerged as promising prebiotics and have been extensively studied. PHFs contain synergistic combinations of botanicals with established anti-inflammatory, antioxidant, and insulin-sensitizing effects (13, 14). These studies highlight a range of beneficial effects, including increased levels of gut commensals, reduction of opportunistic pathobionts, modulation of gut dysbiosis, decreased intestinal inflammation, and hypoglycemic, antioxidant, and insulin-sensitizing properties. For instance, among various plant-based options, several individual herbs have been shown in preclinical and clinical studies to increase beneficial taxa, such as *Bifidobacterium*, suppress gut inflammation, and improve metabolic profiles (15, 16, 17). *Phyllanthus emblica* (also known as *Emblica officinalis*) supports the growth of beneficial bacteria, such as *Bifidobacterium* and *Lactobacillus*, while reducing harmful species, such as *Ruminococcus spp*. and *Clostridium spp*. It has shown prebiotic activity, reduces gut inflammation, and helps modulate dysbiosis (18, 19). Similarly, *Curcuma longa* enhances levels of beneficial microbiota, including genera *Bifidobacterium* and *Lactobacillus*, and reduces populations of opportunistic bacteria belonging to the families *Prevotellaceae* and *Enterobacteriaceae*, and the order *Coriobacterales*, thereby exhibiting strong anti-inflammatory and antioxidant effects (20). In rodent models, supplementation with *Zingiber officinale* increased the abundance of beneficial gut bacteria, including the families *Lactobacillaceae*, *Bacteroidaceae*, and species within the genus *Bifidobacterium*, while reducing potentially pathogenic taxa, such as the families *Clostridiaceae*, *Ruminococcaceae*, and *Desulfovibrionaceae* (21). Finally, *Piper nigrum* supports the growth of beneficial gut flora, such as genera *Bifidobacterium* and *Lactobacillus*, while reducing the relative abundance of phylum *Firmicutes* and *Bacteroides*, showing both prebiotic and anti-inflammatory properties (22). Collectively, these herbs contribute meaningfully to the modulation of gut microbiota and offer therapeutic potential for maintaining gastrointestinal health.

Despite these advances, robust clinical trials evaluating PHFs in metformin-intolerant T2D patients remain scarce. Such studies should ideally incorporate high-resolution metagenomic profiling, continuous glucose monitoring (CGM), and standardized assessment of GI symptoms. Importantly, gut microbiome-based predictive tools for T2D response and tolerability must be tailored to the patient’s age and geographical context (23).

In this randomized-controlled trial, we assessed the short-term effects of a PHF – comprising the previously described herbs (details in the ‘Methods’ section) – co-administered with metformin in T2D patients with prior metformin-related GI intolerance. We hypothesized that PHF would beneficially modulate gut microbial composition and function, enhancing tolerability without compromising glycemic control. While this is the overarching aim of the ongoing clinical trial, this article presents preliminary data supporting this hypothesis. By integrating shotgun metagenomics, CGM, and clinical assessments, this trial seeks to generate translational insights into the role of PHFs as adjuncts in modern, personalized diabetes care.

## Methods

### Study design

This study is a randomized, placebo-controlled, crossover clinical trial registered at ClinicalTrials.gov (ID: NCT06846138). This article reports the preliminary results from the initial 7-day intervention phase, corresponding to the first treatment period only, during which each participant received either PHF or placebo (but not both). The full 48-week crossover analysis will be reported upon study completion. It was designed to evaluate the effects of an add-on polyherbal formulation on gut microbiota composition, functional pathways, and glycemic outcomes and side effects in patients with T2D undergoing metformin therapy. The study protocol was approved by an institutional ethics committee (Approval No. 061124-1L). All study procedures adhered to the principles outlined in the Declaration of Helsinki and followed good clinical practice (ICH-GCP) guidelines.

The ongoing clinical trial is conducted in a certified clinical environment, with sample processing, metagenomic sequencing, and multi-omics analyses performed in accredited research facilities using standardized protocols. The full trial consists of six clinical visits over a 48-week period, with the current report focusing on the short-term outcomes from the first 7-day intervention phase.

### Patient recruitment

Between April and June 2025, a total of 30 participants aged 25–80 years were recruited. All patients had a confirmed diagnosis of T2D according to the WHO criteria and a history of documented or self-reported symptoms of gastrointestinal intolerance to metformin. All participants were maintained on their maximum individually tolerable metformin dose, predominantly as extended-release formulations, reflecting standard practice for patients with prior immediate-release-related gastrointestinal intolerance. Additional inclusion criteria consisted of HbA1c between 6.5 and 8.5%, ongoing stable oral antidiabetic therapy (metformin monotherapy or metformin combination with DPP-4 inhibitors or SGLT2 inhibitors) for at least 6 months. Participants had to be willing and able to provide written informed consent.

Individuals were excluded if they had type 1 diabetes, chronic kidney disease stage IIIb or higher, recent cardiovascular events, such as stroke or myocardial infarction, within the past 6 months, severe diabetic complications, or any active infections requiring antibiotic therapy. Pregnant or lactating women were excluded, as were individuals who had used any polyherbal supplements or probiotics within the 6 months before enrollment. Three participants were excluded from the preliminary analysis: two due to the use of non-metformin antidiabetic therapies and one who exhibited extreme outlier patterns in gut microbiome composition. The latter deviation was likely related to the participant’s extensive use of psychotropic medications – drug classes known to influence gut microbiome composition.

### Randomization and intervention

Randomization was performed using a computer-generated sequence in a 1:1 ratio to allocate participants into one of two treatment sequences: sequence A received PHF first, followed by placebo (*n* = 14), while sequence B received placebo first, followed by PHF (*n* = 13). Each participant completed a 7-day intervention phase, during which blood and stool samples were collected before and after the period. While the full trial is designed to span 48 weeks in a crossover design – with two 24-week treatment periods per participant – this report focuses on the initial 7-day intervention. Both the PHF and placebo capsules were identical in size, color, weight, and appearance to maintain blinding.

The investigational product was a polyherbal formulation provided in 718 mg capsules and produced by a GMP-certified manufacturer. Each capsule contained a proprietary, standardized blend of authenticated herbal extracts with previously reported gut microbiota – modulating and anti-inflammatory properties (the detailed composition is provided in Supplementary Table S7 (see the section on Supplementary materials given at the end of the article)). The placebo capsules matched the PHF in appearance and composition of excipients and were produced under identical manufacturing and quality control protocols to ensure inertness and safety.

Participants received either PHF or placebo in combination with their prescribed extended-release metformin regimen. The short-term microbiome and glycemic monitoring phase of the trial spanned 7 days per arm, with longer-term clinical and metabolic follow-up continuing for 24 weeks per arm (data to be reported separately). Participants were instructed to take three capsules before breakfast and three before dinner throughout the 7-day intervention while maintaining their existing metformin dose. Adherence was assessed at the day 8 follow-up visit using capsule count verification and a structured self-report questionnaire on dosing compliance and gastrointestinal symptoms; no major deviations were reported.

The short-term intervention phase comprised three key visits. At the baseline visit (day 0), eligibility was confirmed, written informed consent was obtained, and anthropometric and clinical data were collected. CGM devices were distributed, and baseline stool and blood samples were collected. On day 1, participants began the assigned intervention (PHF or placebo) in combination with their existing metformin dose. Instructions for capsule administration and sample collection were provided. At the follow-up visit on day 8, CGM sensors were removed, post-intervention stool and blood samples were collected, and participants completed a tolerability questionnaire documenting any gastrointestinal symptoms experienced during the intervention. This same visit structure will be repeated in the second phase of the crossover, beginning at week 24.

### Biological sample collection and processing

Blood samples for biochemical analyses were collected in a clinical laboratory by certified personnel following standard procedures. Participants collected stool samples at home using standardized sterile kits containing nucleic acid preservative buffers (Copan eNAT system, Italy). Samples were stored at −20°C for short-term and at −80°C for long-term storage until analysis. DNA extraction was performed with the MGIEasy Stool Microbiome DNA Extraction Kit on the MGISP-960 automated platform (MGI Tech Co., Ltd, China). The quality and quantity of extracted nucleic acids were assessed using the Agilent 4200 TapeStation System (Agilent Technologies, USA) and the Qubit 4 Fluorometer (Thermo Fisher Scientific, USA), respectively.

Library preparation was performed with the MGIEasy Universal DNA Library Prep Set (MGI Tech Co., Ltd, China), followed by sequencing on the DNBSEQ-G400RS platform (MGI Tech Co., Ltd, China), using DNBSEQ-G400RS high-throughput sequencing set (FCL PE150) (MGI Tech Co., Ltd, China) and obtaining paired-end 150 bp reads with a target depth of 20 million reads per sample.

### Gut microbiome data analysis

Sequencing data underwent quality control with FastQC and Trimmomatic; human DNA contamination was removed using Bowtie2. Taxonomic profiling was performed with Kraken2 and Bracken against the NCBI RefSeq database. Functional pathway profiling was conducted using HUMAnN3 with pathway annotations from the MetaCyc database.

Alpha diversity (Shannon index) was calculated with the vegan package (v2.6.10) in R (v4.3.3), and statistical comparisons were made using the Wilcoxon rank-sum test. Beta diversity was assessed using Bray–Curtis dissimilarities, and the influence of explanatory variables on microbial community composition was analyzed using canonical correspondence analysis (CCA) in R (v4.3.3). Ordinations in this article were constrained by arm and time, the primary variables of interest identified *a priori*. Additional ordinations, adjusting for potential covariates (age, body mass index (BMI), sex, and sequencing batch), were provided in Supplementary Figs S2 and S3. Permutational multivariate analysis of variance (PERMANOVA) based on the Bray–Curtis distances was applied using the adonis2 function from the vegan package in R (v4.3.3) to identify significant factors explaining microbial variance. Differential abundance analyses were conducted using MaAsLin2 (v1.15.1) in R (v4.3.3), adjusting for sex, age, BMI, diabetes duration, metformin dose, and sequencing batch; arm was included for cross-sectional models, and patient ID was incorporated as a random effect for longitudinal analyses. All covariates were included irrespective of their univariate significance because they represent known biological or technical sources of variation in microbiome composition. Taxa were filtered by ≥10% prevalence and ≥0.01 relative abundance, whereas pathways were filtered by prevalence only, since pathway abundances are inherently low and an abundance threshold would remove biologically relevant features. The default false discovery rate (FDR) threshold used in MaAsLin2 and also other microbiome studies is <0.25. This relatively permissive threshold reflects the high dimensionality and inherent variability of microbiome data, where stringent cutoffs may overlook biologically relevant associations. Taxon-level associations with FDR-adjusted *q*-values below 0.25 are therefore commonly interpreted as statistically significant, while still requiring independent validation to confirm their biological relevance, which will be conducted during the upcoming phases of this clinical trial (24, 25, 26). All visualizations were generated using ggplot2 in R (v4.3.3).

### Continuous glucose monitoring

Participants were equipped with Dexcom ONE + CGM sensors, which were applied at the initiation of the intervention (day 1) and removed on day 8. The devices recorded real-time interstitial glucose concentrations every 5 min over the 7-day period. Glycemic parameters assessed included average glucose levels, the glucose management indicator (GMI), coefficient of variation (CV%), time in range (TIR, defined as 3.9–10.0 mmol/L), time above range (TAR, >10.0 mmol/L), and time below range (TBR, <3.9 mmol/L). All CGM data were reviewed for technical integrity, and any datasets from malfunctioning or incomplete sensors were excluded from analysis.

### Statistical analysis of clinical data

Statistical analysis of clinical, laboratory, and CGM data was performed considering a significance level of *P* ≤ 0.05, which was deemed statistically significant. Descriptive statistics were applied to all study variables. Qualitative variables were represented as numbers and percentages of the total study sample. The normality of study variables was assessed using the one-sample Kolmogorov–Smirnov test, and data distribution was visually represented with histograms. Given the non-normal distribution of most of the continuous variables, we reported the median values and interquartile ranges (Q25; Q75). In particular, the Wilcoxon rank-sum test and Chi-squared test were employed for the comparison of different anthropometric and biochemical data involving two or more groups, respectively.

## Results

### Study participants and randomization

Twenty-seven participants with T2D and a history of metformin intolerance were randomized 1:1 to receive either PHF and metformin or placebo and metformin for a 7-day intervention phase. The demographic and clinical characteristics of the two groups were well balanced at baseline (Table 1). No statistically significant differences were observed in age, sex distribution, BMI, diabetes duration, or prevalence of comorbidities, supporting successful randomization.

**Table 1:** Baseline demographic and clinical characteristics.

Variable	PHF group (*n* = 13)	Placebo group (*n* = 14)	*P*-value
Age (years), median (IQR)	68 (61–69)	62.5 (45.5–68.5)	2.74E−01
Female, *n* (%)	10 (76.9%)	10 (71.4%)	1.00E+00
BMI (kg/m^2^), median (IQR)	33.98 (29.94–35.43)	34.33 (29.84–38.74)	9.35E−01
Diabetes duration (years), median (IQR)	5 (4–14)	6 (3–6)	7.83E−01
Cardiovascular disease, *n* (%)	2 (15.4%)	4 (28.6%)	6.48E−01
GI disease, *n* (%)	4 (30.8%)	6 (42.9%)	6.95E−01

PHF, polyherbal formulation; IQR, interquartile range; BMI, body mass index; GI, gastrointestinal.

No significant differences were observed in renal, hepatic, or metabolic parameters at baseline. Of note, calcium levels were lower in the placebo group (*P* = 0.049), but all values remained within reference ranges (Table 2).

**Table 2:** Selected biochemical parameters at baseline (median (IQR)).

Parameter	PHF group (*n* = 13)	Placebo group (*n* = 14)	*P*-value
HbA1c (%)	6.12 (5.62–6.41)	6.05 (5.88–6.20)	8.65E−01
Fasting glucose (mmol/L)	6.22 (5.29–6.48)	6.21 (5.72–6.81)	6.62E−01
Ferritin (ng/mL)	117 (48.1–189)	112.2 (66.7–178.25)	8.84E−01
ALAT (U/L)	25 (21–32)	25.5 (21.3–36.8)	9.81E−01
Creatinine (μmol/L)	67.9 (61.1–86.8)	71.4 (66.3–78.22)	5.93E−01
eGFR (CKD-EPI, mL/min/1.73 m^2^)	85 (84–96)	96 (79.5–99.8)	4.52E−01
Calcium (mmol/L)	2.41 (2.36–2.49)	2.33 (2.31–2.36)	4.90E−02
Magnesium (mmol/L)	0.78 (0.74–0.81)	0.78 (0.73–0.8)	4.96E−01
Cholesterol (mmol/L)	4.61 (3.44–5.12)	4.16 (3.67–5.02)	9.61E−01

PHF, polyherbal formulation; ALAT: alanine aminotransferase; eGFR: estimated glomerular filtration rate.

In the first recruitment block, seven out of 27 participants – five in the PHF group and two in the placebo group – reported mild gastrointestinal symptoms associated with the intervention. These included bloating or meteorism, reported by five participants, and loose stools (1–3 episodes) reported by three participants. All symptoms appeared within the first 4 days of treatment. None of the side effects interfered with daily activities or led to discontinuation. No serious adverse events occurred during the 7-day intervention. These findings will be further explored in subsequent phases of the trial with a larger sample size and extended follow-up.

CGM was successfully deployed in 22 of the 27 participants; however, four datasets were excluded due to incomplete data capture, resulting in 18 participants (nine PHF and nine placebo) included in the final CGM analyses. No statistically significant improvements in CGM-related outcomes were observed during the 7-day intervention in the PHF group (Table 3).

**Table 3:** CGM metrics after 7-day intervention.

Metric	Placebo group (*n* = 9)	PHF group (*n* = 9)	*P*-value
Average glucose (mmol/L)	7.3 (6.25–7.8)	6.6 (5.85–7.05)	1.35E−01
GMI (%)	6.5 (6.0–6.65)	6.2 (5.85–6.4)	1.86E−01
Coefficient of variation (%)	17.2 (14.9–22.5)	16.2 (15.0–17.35)	1.55E−01
TIR (%)	97 (87–98.5)	99 (92–99.5)	1.87E−01
TAR (%)	3 (1–13)	1 (0–5)	8.90E−02
TBR (%)	0 (0–0.5)	0 (0–0.5)	4.76E−01

PHF, polyherbal formulation; GMI, glucose management indicator; TIR, time in range; TAR, time above range; TBR, time below range.

### Microbiome composition and diversity

A total of 54 stool samples were collected from 27 T2D patients treated with metformin (13 in the PHF group and 14 in the placebo group) before and after a 7-day intervention. Shotgun sequencing yielded a median of 19,304,844 reads per sample (IQR: 17,025,742–20,794,100), with 89% of reads classified across 788 species-level taxa. Four comparisons were performed to assess both between-group differences and within-group changes over the 7-day intervention: i) placebo vs PHF group before the intervention, ii) placebo vs PHF group after the intervention, iii) within the placebo group – post-intervention vs pre-intervention samples, and iv) within the PHF group – post-intervention vs pre-intervention samples.

**Figure 1:**
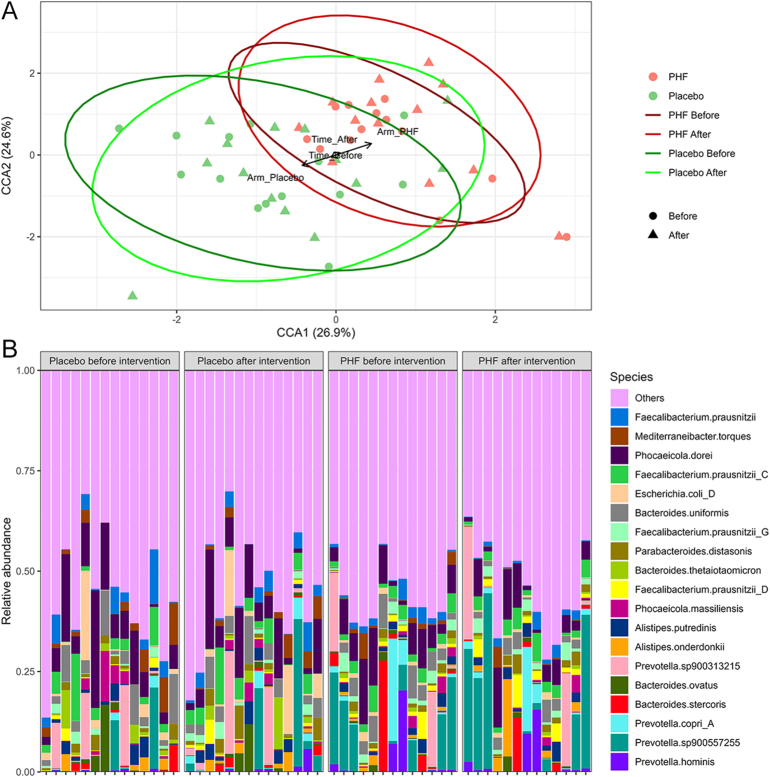
CCA and taxonomic composition of the gut microbiome. (A) CCA of gut microbiome profiles in PHF-treated patients (salmon) and the placebo group (pale green) before and after the 7-day intervention. Time points are distinguished by shape: circles indicate the baseline (before intervention) and triangles indicate the post-intervention period. The colored ellipses represent grouping by study arm (PHF or placebo) and time (samples collected pre- or post-intervention). The arrows represent the centroids of the constrained factors (arm and time) in the CCA model. Only arm was identified as a significant contributor to microbiome variation by PERMANOVA; time is shown for visualization. (B) Taxonomic composition of the 20 most abundant bacterial species across all samples, shown as relative abundances in stacked bar plots. The samples are grouped by study arm and time (placebo group before intervention, placebo group after intervention, polyherbal formulation group before intervention, and polyherbal formulation group after intervention), with taxa ordered by the mean abundance within each group. CCA: canonical correspondence analysis; PHF: polyherbal formulation.

Measures of intra-sample variability, expressed as the Shannon index, showed no statistically significant differences in any of the contrasts analyzed (Supplementary Fig. S1, Supplementary Table S1). To assess the influence of environmental variables on gut microbiome composition, CCA combined with permutational multivariate analysis of variance (PERMANOVA) was applied (Fig. 1A, Supplementary Figs S2 and S3). PERMANOVA identified study arm as a significant factor explaining variation in gut microbiome composition (*R*^2^ = 0.04; *P* = 4.01E-02), along with patient identity (*R*^2^ = 0.88; *P* = 1.00E-04) (Supplementary Table S2).

*Phocaeicola dorei* was the most abundant species in the placebo group at both baseline (9.27%) and after the intervention (8.11%). In the PHF group, however, *P. dorei* was not the dominant species at either time point. At baseline, *Prevotella sp900557255* was already more abundant (7.77%) than *P. dorei* (7.11%). Following the 7-day intervention, this difference became more pronounced, with *Prevotella sp900557255* increasing to 11.40%, while *P. dorei* decreased to 5.54% (Fig. 1B).

**Figure 2:**
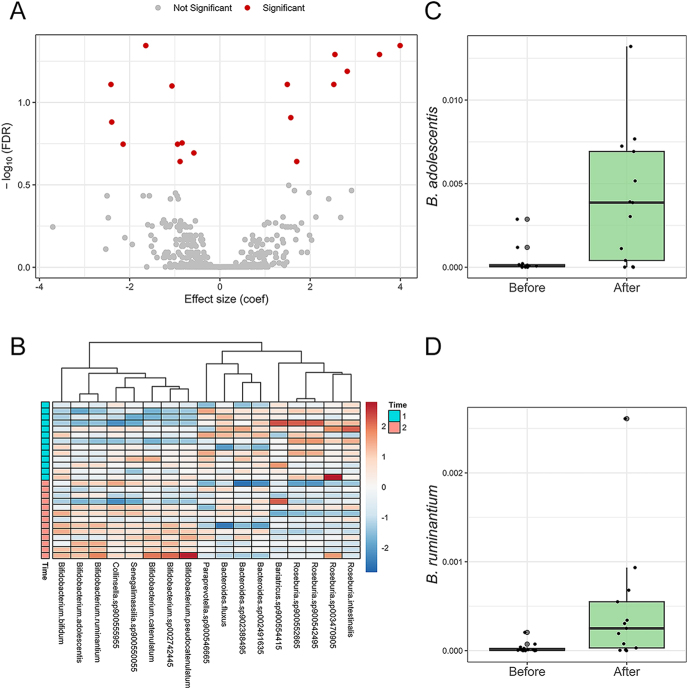
Changes in gut microbiota associated with polyherbal formulation intake during the intervention. (A) Volcano plot showing species associated with polyherbal formulation intake in the longitudinal comparison (post- vs pre-intervention) within the PHF arm (FDR < 0.25). (B) Heatmap of relative abundances (*z*-scored) of the significantly associated species (FDR < 0.25) in PHF arm samples at baseline (time 1) and after the intervention (time 2). (C) Relative abundance of *Bifidobacterium adolescentis* before and after PHF intake. (D) Relative abundance of *Bifidobacterium ruminantium* before and after PHF intake. Boxplots (C and D) show individual data points and box interquartile ranges. PHF: polyherbal formulation; FDR: false discovery rate.

Among the four comparisons performed – i) placebo vs PHF group before the intervention, ii) placebo vs PHF group after the intervention, iii) within the placebo group (post-intervention vs pre-intervention), and iv) within the PHF group (post-intervention vs pre-intervention) – only the longitudinal comparison within the PHF group revealed statistically significant differences (FDR < 0.25). In total, 17 taxa exhibited significant changes in abundance during the 7-day intervention in the PHF group (Fig. 2A and B). Among these, all six detected *Bifidobacterium* species increased, with the strongest associations observed for *B. adolescentis* (coef. = 3.99, FDR = 4.52E-02) and *B. ruminantium* (coef. = 3.54, FDR = 5.12E-02) (Fig. 2C and D). Among the taxa that decreased, *Roseburia intestinalis* (coef. = −2.42, FDR = 7.78E-02) and *Roseburia sp900552665* (coef. = −2.40, FDR = 1.32-01) showed the strongest negative associations (Supplementary Table S3).

### Functional analysis

**Figure 3:**
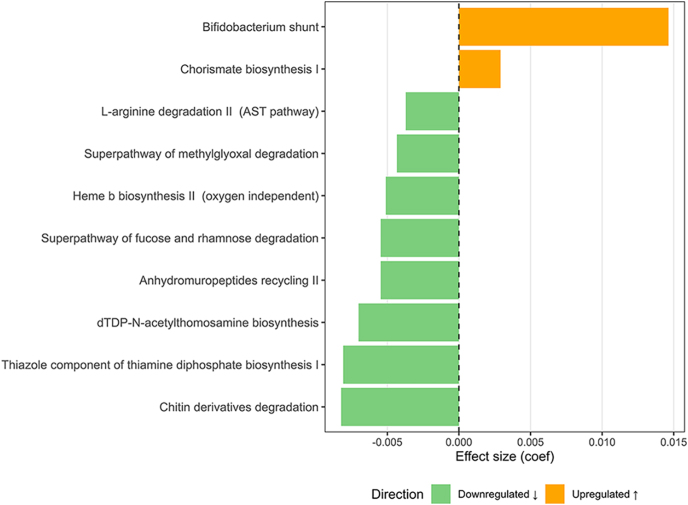
Differentially abundant MetaCyc pathways in the polyherbal formulation group when comparing post-intervention samples to pre-intervention samples. The bar plot shows the MaAsLin2-derived effect sizes (coefficients) for MetaCyc pathways that changed significantly (FDR < 0.25) after the intervention in the PHF group. Positive coefficients indicate higher pathway abundance post-intervention, whereas negative coefficients indicate lower abundance post-intervention. PHF: polyherbal formulation; FDR: false discovery rate.

The functional profiling of the gut microbiome, based on gene family-derived MetaCyc pathway abundances, was assessed using the same comparison groups as in the taxonomic analyses. To identify key factors explaining the variance in microbial pathway composition, we applied PERMANOVA, which revealed that each individual patient (*R*^2^ = 0.85, *P* = 1.00E-04), study arm (PHF vs placebo) (*R*^2^ = 0.07, *P* = 1.28E-02) and sex (*R*^2^ = 0.06, *P* = 2.64E-02) significantly contributed to the variation (Supplementary Table S4). Differential abundance analysis identified ten pathways significantly associated with PHF intake (FDR < 0.25), with eight showing decreased abundance – for example, the methylglyoxal degradation pathway (METHGLYUT-PWY, coef. = −0.0043, FDR = 9.92E-02) – and two showing increased abundance, including the *Bifidobacterium* shunt (P124-PWY, coef. = 0.0146, FDR = 2.24E-01) and Chorismate biosynthesis I (ARO-PWY, coef. = 0.0029, FDR = 2.27E-01) (Fig. 3, Supplementary Table S5). In the placebo group, a small but significant increase was observed in the incomplete reductive tricarboxylic acid (TCA) cycle pathway (P42-PWY, coef. = 0.0061, FDR = 2.27E-01) (Supplementary Table S6).

## Discussion

This preliminary analysis of the first 7-day intervention phase of a randomized, placebo-controlled crossover trial provides evidence that a polyherbal formulation co-administered with metformin can rapidly and selectively modulate the gut microbiome of individuals with T2D who experience gastrointestinal intolerance to metformin. Although glycemic metrics remained unchanged after a short-term intervention, the significant compositional and functional shifts in the microbiota – particularly the enrichment of *Bifidobacterium* spp. – suggest a promising microbiome-targeted approach for enhancing the tolerability and potentially the long-term efficacy of metformin therapy (3, 8).

Consistent with prior studies on the gut microbiome modulatory effects of PHF, we did not observe any significant changes in alpha diversity but noted a significant shift in beta diversity in the PHF group after the 7-day intervention (4, 7). Importantly, our metagenomic analysis revealed the selective enrichment of six *Bifidobacterium* species, including *B. adolescentis* (coef. = 3.99; FDR = 4.52E-02) and *B. ruminantium* (coef. = 3.54; FDR = 5.12E-02), both of which have been associated with anti-inflammatory effects, improved intestinal barrier function, and beneficial carbohydrate metabolism (27, 28). These microbial shifts occurred in the absence of similar changes in the placebo group, suggesting a direct effect of PHF on gut ecology. The enrichment of *Bifidobacterium* may be mechanistically significant. Several strains within this genus are known to produce acetate and lactate, lowering luminal pH, which can inhibit the growth of pathobionts and promote mucosal integrity (28). Moreover, *Bifidobacterium spp*. can degrade complex plant-derived polysaccharides, indicating that PHF may act via a prebiotic-like mechanism, selectively fueling beneficial taxa (10, 29). This aligns with other reports, where PHF enhanced microbial richness and host tolerance to antidiabetic drugs (14, 15). Notably, the functional relevance of increased *Bifidobacterium* abundance is further supported by our pathway analysis, which showed a significant increase in the *Bifidobacterium* shunt metabolic pathway in the PHF group during the 7-day intervention. The *Bifidobacterium* shunt, also known as the fructose-6-phosphate phosphoketolase pathway, is a distinctive metabolic route in *Bifidobacterium* species that enables efficient carbohydrate utilization and leads to the production of beneficial short-chain fatty acids (SCFAs), such as acetate and lactate. These SCFAs contribute to gut health by supporting epithelial integrity, modulating immune responses, and inhibiting pathogenic microbes (30). This functional evidence strengthens the hypothesis that PHF exerts its effects, at least in part, through promoting beneficial microbial activity within the gut ecosystem.

Interestingly, we observed a significant decrease in several *Roseburia* species during the PHF intervention, including *Roseburia intestinalis* and *Roseburia sp900552665* (coefficients ranging from −2.42 to −1.64 accordingly; FDR < 0.25). Although *Roseburia* is a butyrate-producing genus generally linked to gut health, recent studies have shown a negative association between *Roseburia* abundance and T2D (31). Furthermore, metformin therapy has been previously reported to reduce *Roseburia* levels in T2D patients (32, 33). Our findings suggest that PHF may enhance this metformin-associated decline in *Roseburia*, potentially amplifying metformin’s impact on gut microbiota composition. While the functional significance of *Roseburia* reduction remains uncertain, these results highlight the complexity of host–microbiome–drug interactions and imply that the role of butyrate producers, such as *Roseburia*, may be context-dependent in T2D. Further studies are warranted to determine whether this decline contributes to PHF’s clinical effects or reflects a specific microbial adaptation to the combined treatment.

In addition, we observed a significant, although mild, increase in the abundance of *Collinsella sp900555955* during the PHF intervention (coef. = 1.57, FDR = 1.24E-01). The genus *Collinsella* has been controversially linked to metabolic disturbances, such as gestational diabetes mellitus, increased circulating insulin levels, and low dietary fiber intake (34, 35). In obese pregnant women, higher fiber intake was associated with reduced *Collinsella* abundance – a change considered beneficial (35). These associations seem to conflict with the hypothesis that PHF promotes a healthier gut microbiome composition. However, previous studies relied on 16S rRNA gene sequencing, offering only genus-level resolution (35, 36), whereas our shotgun metagenomic approach provides species-level insights. This higher resolution may uncover species-specific functional traits within the *Collinsella* genus that were not detected in earlier studies. The observed increase in *Collinsella sp900555955* may thus reflect distinct ecological roles or metabolic activities, highlighting the importance of species-level investigations in understanding host–microbiome interactions in metabolic disease contexts.

In addition to the increased abundance of the *Bifidobacterium* shunt pathway in the PHF group during the 7-day intervention, our analysis revealed differences in several other microbial metabolic pathways due to the administration of PHF. These included a lower abundance of the methylglyoxal degradation and anhydromuropeptide recycling pathways. Given that dysregulated methylglyoxal (MG) metabolism and impaired glyoxalase activity in human tissues are strongly implicated in the development of diabetes and its complications (37), emerging evidence that gut microbes also possess MG-detoxifying pathways capable of modulating host carbonyl stress (38) highlights the potential relevance of the observed changes in the microbial methylglyoxal degradation pathway in our cohort. In contrast, the anhydromuropeptide recycling pathway reflects how bacteria break down and rebuild their cell wall, a process that releases small muropeptide fragments. These fragments can activate host immune sensors, such as nucleotide-binding oligomerization domain-like (NOD) receptors, and thereby influence intestinal inflammatory responses (39). Nevertheless, as these findings are based on gene family abundance predictions rather than direct metabolite measurements, they remain indirect and require validation – an aim to be addressed in the ongoing trial through planned stool metabolomics analyses.

Despite notable microbiota changes, no significant differences in glycemic metrics were observed between PHF and placebo groups during the 7-day intervention (Table 3). This aligns with the expected pharmacokinetics of metformin and its mechanisms of glucose lowering, which generally require several weeks to manifest fully (40). Nonetheless, there was a non-significant trend toward lower average glucose and TAR in the PHF group. These early patterns, if confirmed in the ongoing longer-term arm of the trial, could suggest a potential synergistic effect between PHF and metformin in improving glucose control. However, short duration and small sample size preclude definitive conclusions at this stage.

Metformin remains the cornerstone of T2D management worldwide; however, 30–50% of patients experience gastrointestinal side effects, and nearly half discontinue therapy over the long term (1, 16). Identifying strategies to mitigate these adverse effects is essential for improving treatment adherence and outcomes. In this first-in-human study, we observed that a standardized, GMP-certified polyherbal formulation – already registered for clinical use – induced shifts in the gut microbiota that may be associated with improved gastrointestinal health over time. While these microbial changes are promising, their clinical relevance remains uncertain, as no improvement in glycemic control or gastrointestinal side effects was observed within the short intervention period. Importantly, the long-term clinical hypothesis that will be tested in this crossover study is that by favorably shifting the gut microbiome, PHF could reduce metformin-related gastrointestinal intolerance and potentially enhance its glycemic efficacy. Given its multi-herbal composition, PHF may exert broad-spectrum modulatory effects on the gut microbiome, potentially complementing existing approaches, such as probiotics or prebiotics (13). In addition, its oral administration and lack of systemic pharmacodynamic targets suggest a low risk of adverse interactions, which may be particularly advantageous in elderly or comorbid T2D patients. However, its role as an adjunct to metformin therapy requires further validation in longer-term studies with clinical endpoints.

This interim analysis has several limitations that must be acknowledged. First, the short intervention duration of 7 days, while adequate to observe early microbial shifts, is insufficient to assess long-term clinical or metabolic outcomes. Second, the sample size, although powered for microbiome endpoints, limits broader generalizability and may not capture smaller glycemic effects. Third, malfunction or incomplete data from 20% of CGM devices reduced statistical power to detect differences in glycemic variability. Fourth, the study did not employ a validated gastrointestinal symptom questionnaire, relying instead on qualitative participant reports. This will be rectified in subsequent phases with structured symptom scoring and stool metabolomics. Fifth, participants followed habitual diets with only partial dietary monitoring through food diaries, which introduces potential confounding from uncontrolled dietary intake. Finally, we observed slightly lower baseline blood calcium levels in the placebo group compared to the PHF group (2.33 vs 2.41 mmol/L; *P* = 0.049); however, after reviewing comorbidities, this difference was considered clinically insignificant.

In this initial phase of a larger clinical trial, a 7-day course of PHF added to metformin significantly altered the gut microbiome in patients with T2D, notably increasing the abundance of *Bifidobacterium* species. These shifts occurred without compromising glycemic control but, at this stage, cannot be directly linked to clinical benefit, including improved gastrointestinal tolerability. While these early findings suggest that PHF may have potential as a microbiome-modulating adjunct to metformin, further validation is needed. The ongoing 48-week crossover trial will provide long-term clinical and microbiome data, along with stool metabolomics, to better understand functional implications and potential therapeutic benefits of PHF co-administration in T2D. In particular, it will evaluate whether favorable modulation of the gut microbiome by PHF can mitigate metformin-related gastrointestinal side effects and enhance its glycemic efficacy.

## Supplementary materials





## Declaration of interest

The authors declare that there is no conflict of interest that could be perceived as prejudicing the impartiality of the work reported.

## Funding

Funded by the Latvian Council of Science (Fundamental and Applied Research Project No. lzp-2023/1-0422 ‘MetHerb: Enhancing the efficacy and tolerability of metformin in the analysis of gut microbiome by add-on polyherbal formulation’).

## Data availability

Metagenomic data have been deposited in the European Nucleotide Archive (ENA).

## Ethics approval

Approved by the Ethics Committee of Pauls Stradiņš Clinical University Hospital (Approval No. 061124-1L).

## Trial registration

This study is registered at ClinicalTrials.gov (ID: NCT06846138).

## Investigational product

The investigational product Herbomix® was manufactured by Himalaya Nutravedics India Private. The formulation is registered for clinical use by the Latvian Food and Veterinary Agency (Registration No. 16958).
