# Supplementary material for: Rapid and selective gut microbiome modulation by polyherbal formulation in type 2 diabetes
Source: Endocr Connect. 2026 Jan 9;15(1):e250463. doi: 10.1530/EC-25-0463 (PMC12793969; doi:10.1530/EC-25-0463)
Supplement: Supplementary file 1 [file supplementary_figures.pdf]

Supplementary Material

Rapid and Selective Gut Microbiome Modulation by Polyherbal Formulation in Type 2 Diabetes

Sintija Sauša, Annija Zodāne, Somit Kumar, Jānis Plūme, Jana Baranova, Tatjana Kozlova, Uģis Klētnieks, Harijs Saušs, Jānis Kloviņš, Valdis Pīrāgs, Kakarla Sai Mitravinda, Svjatoslavs Kistkins, Monta Brīvība

* Correspondence: **Corresponding Author**: monta@biomed.lu.lv

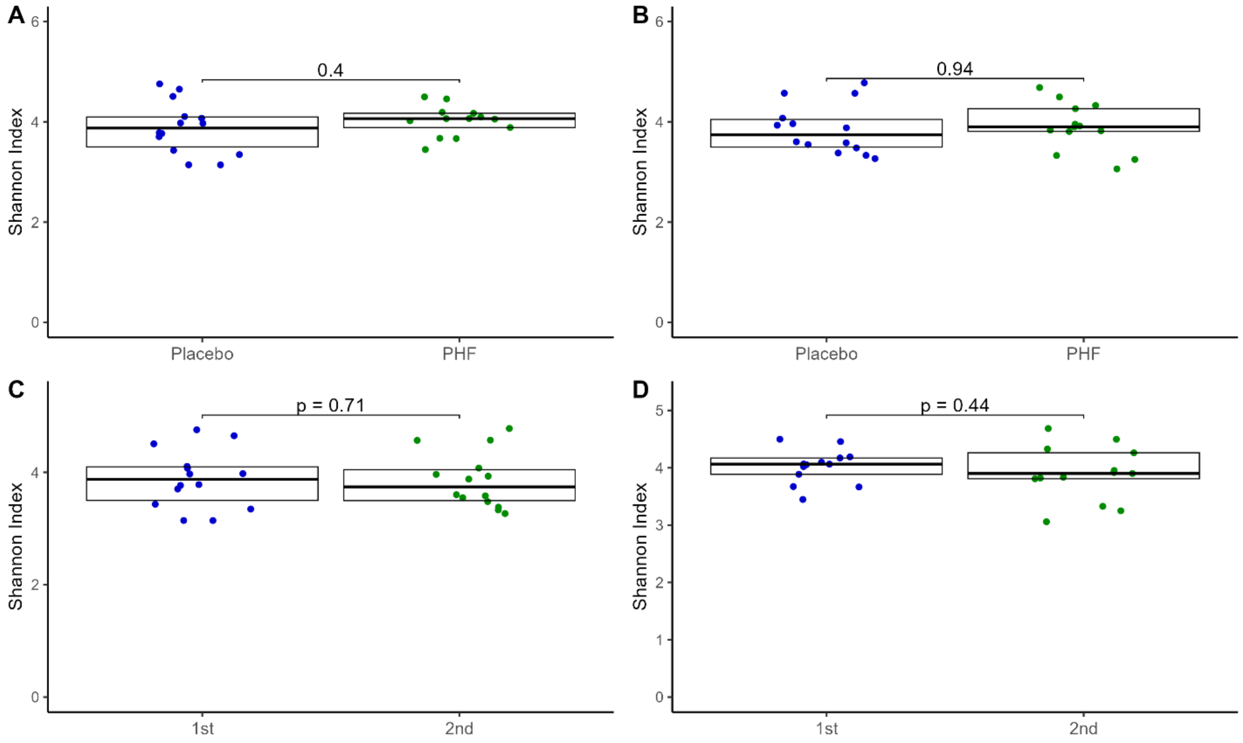

Figure S1. Boxplots of Shannon index comparisons. (A) Placebo vs. PHF groups before the intervention; (B) Placebo vs. PHF groups after the intervention; (C) Placebo group before vs. after the intervention (1st and 2nd visits); (D) PHF group before vs. after the intervention (1st and 2nd visits). P-values from Wilcoxon signed-rank and Wilcoxon rank-sum tests are shown.

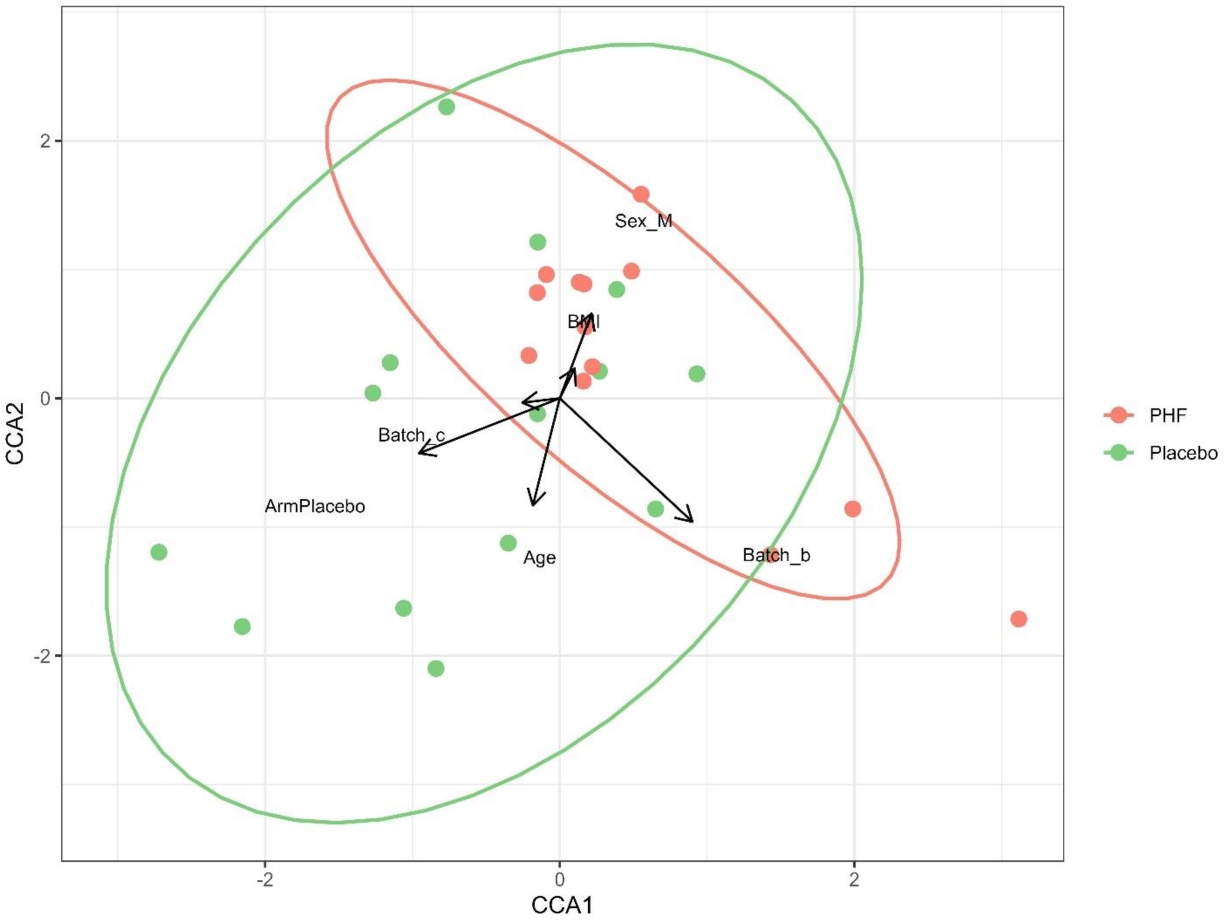

Figure S2. Canonical correspondence analysis (CCA) plot visualizing gut microbiome variation in PHF-treated patients (pastel red) and placebo group (pastel green) before the 7-day intervention. Sample timepoints are coded as follows: Before — samples collected prior to intervention; After — samples collected post-intervention. Arrows represent environmental covariates included in the CCA model; significance of these factors was assessed by PERMANOVA on the full dataset, with treatment arm being the only significant factor.

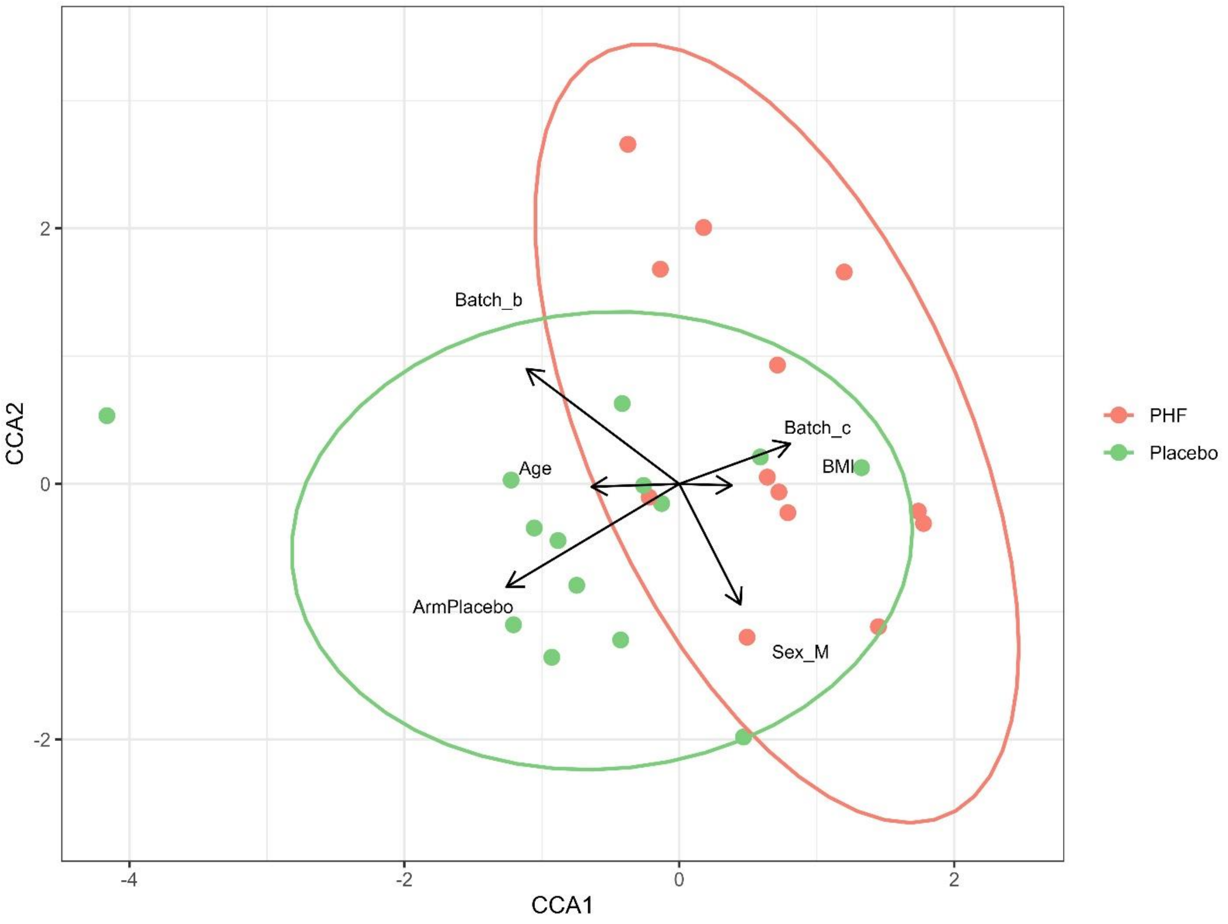

Figure S3. Canonical correspondence analysis (CCA) plot visualizing gut microbiome variation in PHF-treated patients (pastel red) and placebo group (pastel green) after the 7-day intervention. Sample timepoints are coded as follows: Before — samples collected prior to intervention; After — samples collected post-intervention. Arrows represent environmental covariates included in the CCA model; the significance of these factors was assessed by PERMANOVA on the full dataset, with treatment arm being the only significant factor.
